# Effect of Parecoxib as an Adjunct to Patient-Controlled Epidural Analgesia after Abdominal Hysterectomy: A Multicenter, Randomized, Placebo-Controlled Trial

**DOI:** 10.1371/journal.pone.0162589

**Published:** 2016-09-13

**Authors:** Wei-Feng Liu, Hai-Hua Shu, Guo-Dong Zhao, Shu-Ling Peng, Jin-Fang Xiao, Guan-Rong Zhang, Ke-Xuan Liu, Wen-Qi Huang

**Affiliations:** 1 Department of Anesthesiology, The First Affiliated Hospital, Sun Yat-sen University, Guangzhou, China; 2 Department of Anesthesiology, GuangDong General Hospital and GuangDong Academy of Medical Sciences, Guangzhou, China; 3 Department of Anesthesiology, The Second Affiliated Hospital, Sun Yat-sen University, Guangzhou, China; 4 Department of Anesthesiology, NanFang Hospital, Guangzhou, China; 5 Health Management (Examination) Center, GuangDong General Hospital and GuangDong Academy of Medical Sciences, Guangzhou, China; Nanjing University Medical School Affiliated Nanjing Drum Tower Hospital, CHINA

## Abstract

**Objective:**

This multicenter, randomized, placebo-controlled study evaluated the efficacy and side effects of parecoxib during patient-controlled epidural analgesia (PCEA) after abdominal hysterectomy.

**Methods:**

A total of 240 patients who were scheduled for elective abdominal hysterectomy under combined spinal-epidural anesthesia received PCEA plus postoperative intravenous parecoxib 40 mg or saline every 12 h for 48 h after an initial preoperative dose of parecoxib 40 mg or saline. An epidural loading dose of a mixture of 6 mL of 0.25% ropivacaine and 2 mg morphine was administered 30 min before the end of surgery, and PCEA was initiated using 1.25 mg/mL ropivacaine and 0.05 mg/mL morphine with a 2-mL/h background infusion and 2-mL bolus with a 15-min lockout. The primary end point of this study was the quantification of the PCEA-sparing effect of parecoxib.

**Results:**

Demographic data were similar between the two groups. Patients in the parecoxib group received significantly fewer self-administrated boluses (0 (0, 3) vs. 7 (2, 15), *P* < 0.001) and less epidural morphine (5.01 ± 0.44 vs. 5.95 ± 1.29 mg, *P* < 0.001) but experienced greater pain relief compared with the control group (*P* < 0.001). Patient global satisfaction was higher in the parecoxib group than the control group (*P* < 0.001). Length of hospitalization (9.50 ± 2.1, 95% CI 9.12~9.88 vs. 10.41 ± 2.6, 95% CI 9.95~10.87, P = 0.003) and postoperative vomiting (17% vs. 29%, *P* < 0.05) were also reduced in the parecoxib group. There were no serious adverse effects in either group.

**Conclusion:**

Our data suggest that adjunctive parecoxib during PCEA following abdominal hysterectomy is safe and efficacious in reducing pain, requirements of epidural analgesics, and side effects.

**Trial Registration:**

ClinicalTrials.gov (NCT01566669)

## Introduction

Postoperative pain is one of the most important factors that prolongs time to recovery and delays discharge after surgery [1–3]. Patient-controlled epidural analgesia (PCEA) and patient-controlled intravenous analgesia (PCIA) offer good postoperative pain control [4]. Several randomized controlled trials demonstrated that patients treated with a combination of epidural opioids and local anesthetics exhibit improved pain scores and fewer side effects compared with patients treated with PCIA [5, 6]. Opioid therapy is recommended as a first-choice medication for postoperative pain management, but it is associated with several undesirable adverse effects, such as nausea, vomiting, sedation, and respiratory depression [7]. Acute pain service (APS) is important in the provision of effective pain relief and minimizing opioid-related side effects. The presence of APS, like in many other developing countries, is at a young stage in China [8]. Therefore, it is necessary to reduce the need for opioids during the postoperative period. Numerous studies demonstrated that multimodal therapy for postoperative analgesia has advantages over the use of opioids alone [9, 10]. The combination of nonsteroidal anti-inflammatory drugs (NSAIDs) and opioids improved analgesia by inhibiting nociceptive impulses at central and peripheral sites of the pain transmission pathway and reduced the need for opioids during the postoperative period [11]. Selective cyclooxygenase-2 (COX-2) inhibitors (coxibs) reduce postoperative pain without interfering with the normal mechanisms of platelet aggregation and hemostasis or increasing intraoperative blood loss. Therefore, these drugs may demonstrate a higher safety margin than non-selective NSAIDs [12–14].

Previous studies demonstrated that parecoxib sodium, a highly selective COX-2 inhibitor, is effective for the treatment of postoperative pain following various types of surgery [15–18]. Parecoxib was the first clinically available intravenous coxib with a greater analgesic efficacy that may produce synergistic effects with epidural opioids on postoperative pain relief. The present study investigated the effect of parecoxib as an adjuvant to a multimodal PCEA approach in patients undergoing gynecological surgery, with a hypothesis that parecoxib might reduce epidural morphine consumption (presented as a reduction in PCEA boluses).

## Materials and Methods

### Study design and patient selection

This multicenter, randomized, double-blinded, placebo-controlled trial adhered to the CONSORT guidelines for the reporting of randomized trial results [19] (S1 Checklist and S1 and S2 Protocols). The study was registered at ClinicalTrials.gov (NCT01566669) (Prior to the trial, we read many research papers and found only a few trial registrations. Therefore, we didn’t recognize the importance of pre-registering a trial on clinical trials site due to our limited knowledge. By 2012, we realized this problem and made an immediate compensation by registering this trail at ClinicalTrials.gov. Anyway, before and after registering, we're quite sure that the protocols of the trial and its conduction have not been modified.). The authors confirm that all ongoing and related trials for this drug/intervention are registered. The Research Ethics Board at the First Affiliated Hospital, Sun Yat-sen University, approved this study (20090214, Guangzhou, China) on 12 March 2009. Women with an American Society of Anesthesiologists physical status class I-II, aged 18–64 years, who were undergoing abdominal hysterectomy (with or without oophorectomy) under combined spinal-epidural anesthesia (CSEA), were assessed for study eligibility. Patients were recruited on the day before surgery by a co-investigator. Written informed consent was obtained prior to the day of surgery. The study was conducted from June 2009 to May 2010 at four centers in Guangzhou, China (see Appendix).

The exclusion criteria included contraindications for CSE placement; known allergy, sensitivity, or contraindication to opioid and non-opioid analgesic drugs; history of bleeding disorders; peptic ulceration; or anticoagulant use within the past month; drug or alcohol abuse; current pregnancy or breastfeeding; and lack of ability to understand the use of pain assessment scales and the PCA device. Patients with asthma or bronchospasm who required treatment with glucocorticoids, poorly controlled hypertension or diabetes, a chronic or acute renal or hepatic disorder, or inflammatory bowel disease were also excluded. Patients were also excluded if they had used long-acting NSAIDs in the 4 days prior to the first dose of study medication or if they had taken antidepressants, narcotic analgesics, antihistamines, anxiolytics, hypnotics, sedatives, NSAIDs, or corticosteroids up to 24 h before administration of the study medication.

### Randomization and masking

Eligible patients from each hospital were enrolled and received a sequential study number, which allocated them to one of the two study groups according to block randomization of a pre-assigned list. Randomization lists were generated using SAS software and consisted of assignments in blocks of four, with two patients in each block assigned to receive the study drug and the other two patients assigned to the control group. The allocation codes were maintained in pre-prepared opaque envelopes, which were used to label the drug packaging, and disclosed only after a patient was allocated to the next unique participation number. An independent statistician stored the randomization lists and envelopes. Participants, anesthesiologists, and observers were blinded to the treatment assignments. All statistical analyses were performed with masking maintained.

### Study procedures

The patient was placed in the lateral position to receive the CSEA. The back was sterilely prepared and draped, and 1% lidocaine was injected subcutaneously for local anesthesia into the lower lumbar vertebral interspace between L2 and L4. The anesthesiologist who performed the procedure chose the exact injection site after an examination of the patient’s back.

The epidural space was identified using a loss of resistance technique with saline. A non-interlocking 25-gauge Whitacre spinal needle (Becton Dickinson Medical Devices Co. Ltd., Shanghai, China) was advanced through the Weiss needle using the needle-through-needle technique until the hubs touched to reach the subarachnoid space. The presence of spontaneous fluid returning to the spinal needle hub indicated the intrathecal space. The spinal component, which consisted of 10–15 mg of 0.5% or 0.75% hyperbaric bupivacaine, was infused into the intrathecal space. The spinal needle was removed, and a 19-gauge, Flextip Plus^®^ (single open-ended hole) epidural catheter (Arrow International, Inc., USA) was inserted in a cephalad direction and secured with 4–5 cm remaining inside the epidural space. Any evidence of catheter entry into an epidural vein or cerebrospinal fluid excluded the patient from the study. No epidural medications were administered until 15 min after the spinal dose administration. A test dose of 2 mL of 2% lidocaine was administered via the epidural catheter after the initial 15-min period. If the spinal block proved insufficient for surgery, epidural 1% ropivacaine supplements (maximum of 8 mL) were administered as clinically indicated to maintain a T6 level of sensory blockade. Otherwise, the patient underwent general anesthesia and was excluded from the study.

Patients were randomized 1:1 to receive either 40 mg of parecoxib sodium (Pfizer Ltd, Pharmacia and Upjohn Company) (Group Parecoxib) IV or saline (Dazhong Pharmaceuticals Limited, China) (Group Control) at the same volume prior to the initial incision. Thereafter, patients in the parecoxib group received 40 mg of parecoxib IV every 12 h, and patients in the control group received 2 mL of saline every 12 h for 48 h. Post-anesthesia care unit nurses who were blinded to the study prepared the intervention drugs during the study. A mixture of 6 mL of 0.25% ropivacaine and 2 mg morphine was administered epidurally 30 min prior to the end of surgery. PCEA was initiated using 0.125% (1.25 mg/mL) ropivacaine and 0.005% (0.05 mg/mL) morphine with a basal infusion rate of 2 mL/h, a demand dose of 2 mL, and a 15-min lockout. Patients were instructed prior to surgery to use the PCEA mode at their discretion to maintain a numerical rating score (NRS) less than 3. Patients received 1 mg/kg of intravenous tramadol if the NRS was 4 or greater. Tropisetron (0.1 mg/kg) and dexamethasone (0.1 mg/kg) were routinely used for antiemetic prophylaxis 30 min before the end of surgery.

### Primary and secondary outcome measures

The primary end point of this study was the quantification of the PCEA-sparing effect of a multimodal approach that contained parecoxib compared with a standard PCEA approach that did not contain parecoxib in patients undergoing abdominal hysterectomy. The number of PCEA bolus doses and total morphine consumption administered by the patient for 48 h postoperatively were recorded.

Secondary outcomes included pain intensity, the need for rescue analgesics, global satisfactory patient evaluation, and side effects, such as nausea, vomiting, pruritus, sedation, motor block in the lower extremities, and the time to the passage of flatus and the first bowel movement. Pain intensity was recorded on a 100-mm NRS ranging from 0 (no pain) to 100 (worst pain imaginable) and measured at rest and during activity. Pain during activity was elicited by asking the patient to take a deep breath followed by a forceful cough. A pain assessment was obtained immediately after the cough. A global satisfactory evaluation (using a 100-point scale from 1 = highly dissatisfied to 100 = highly satisfied) was obtained from the patient 48 h after skin closure. Assessment of sedation was performed using the Ramsay Sedation Scale (RSS). Motor block was assessed using a modified Bromage scale. Blood loss, length of hospitalization, and postoperative cardiovascular (CV) events were also recorded. The follow-up period was finished after the patient was discharged. Discharge was assessed by the surgeons according to the following discharge criteria: a) pain controlled with oral analgesics; b) stable vital signs; c) afebrile; d) passing flatus; e) incision clean, dry, and intact; and f) full diet tolerated. Pain scores, vital signs (measured using standard monitors during the first 24 h and a portable blood pressure monitor and pulse oximeter 36 h and 48 h after skin closure), the RSS, recovery of gastrointestinal function, and side effects were assessed at 4, 8, 12, 24, 36, and 48 h after skin closure by an investigator who was blinded to the treatment groups.

### Blood test monitoring

Blood samples were drawn before and 48 h after surgery to determine liver (alanine aminotransferase and aspartate amino transferase) and renal (plasma creatinine and blood urea nitrogen concentration) function. Prothrombin time, active partial thromboplastin time, and international normalized ratio (INR) were also assessed.

### Statistical analysis

The primary outcome of the study was PCEA-sparing effect of parecoxib. Coxibs purportedly reduced PCEA boluses in multimodal postoperative analgesia by approximately 32% [20]. We determined the sample size using information about expected reductions in the total self-administered PCEA boluses over 48 h, which was 16.25 ± 12.26 in a pilot observation study, and previous study [20]. These values were entered into the Stata program to calculate the sample size for a two-group trial, with a type-I error rate of 0.05. Accordingly, 100 subjects were required in each group to achieve 80% power to detect a reduction in PCEA boluses by 30%. Assuming a 20% dropout rate, the total number of participants was calculated as 240 (120 per group).

Statistical analyses were conducted on the intent-to-treat (ITT) population (all patients who were randomized and received at least one follow-up) and the efficacy-evaluable (EE) population (all patients who completed the full follow-up) using SPSS 19.0 software (SPSS, Chicago, IL, USA). Demographic and outcome data are presented as frequencies for categorical variables and means ± standard deviations (SD) or medians with quartiles for continuous variables according to patient distributions. Baseline data were analyzed using the t-test, Wilcoxon rank-sum test or chi-square test, as appropriate. General linear models were employed to estimate the mean differences between the parecoxib and placebo groups in medication use, adjusted for the clustering of participating centers. Least-squares means with 95% confidence intervals (CIs) were reported. To estimate mean changes in the NRS from baseline to follow-ups, linear mixed models for repeated measures were used, taking into account the correlations between individuals' repeated measures over time. We also performed sensitivity analyses using the EE population approach to evaluate the impact of attrition after the exclusion of subjects with missing readings at the final follow-up. A *P* value less than 0.05 was considered statistically significant.

## Results

### Patient disposition and baseline characteristics

**Fig 1:**
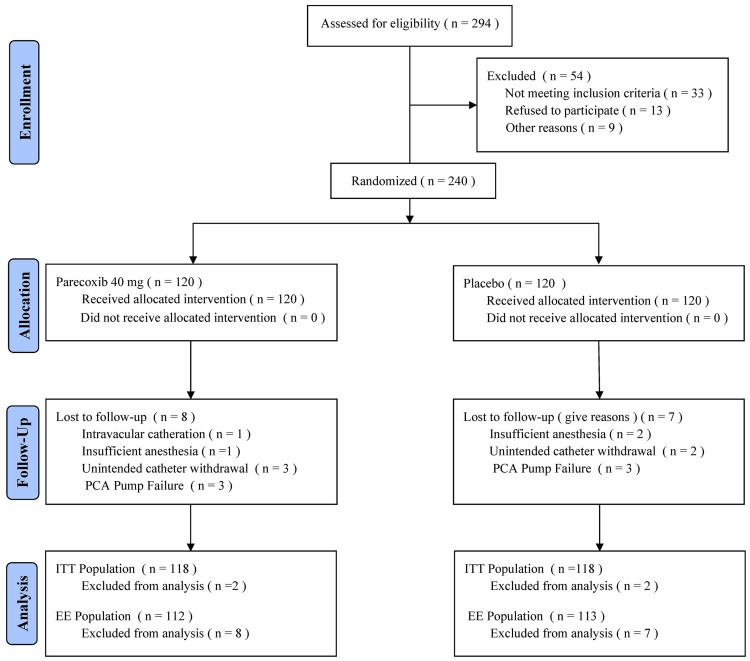
Distribution of patients randomized to receive IV parecoxib or placebo for the management of postoperative pain. PCA = patient-controlled analgesia. ITT = intent-to-treat; EE = efficacy evaluable.

Patient recruitment commenced in June 2009 and concluded in May 2010. Two hundred and ninety four women were assessed for eligibility to enter the study. Fifty-four patients were not eligible or refused to participate in the initial phase of the trial. Therefore, 240 subjects remained, and consent was obtained. These subjects were enrolled and randomized to receive parecoxib (n = 120) or placebo (n = 120). Follow-up evaluations were incomplete in 15 patients (8 in the parecoxib group and 7 in the placebo group). Data from 4 of these patients (2 in each group) were excluded in the final statistical evaluation. Finally, a total of 236 patients were included in the ITT population (118 in each group), and a total of 225 patients were included in the EE population (112 in the parecoxib group and 113 in the placebo group). Fig 1 documents the patient flow throughout the trial.

The groups were comparable with respect to age, body mass index (BMI), ASA physical status, duration of surgery and anesthesia, fluid infusion, blood loss, urinary output, and ropivacaine supplement intake (Table 1). The distribution of each stratum in the two groups was similar.

**Table 1:** Demographic Data and Intraoperative Data.

Item	Parecoxib Group (n = 118)	Control Group (n = 118)	*P* Value
Age (yr)	42 ± 7	42 ± 8	
BMI (kg/m^2^)	22.7 ± 2.6	22.3 ± 2.5	
Duration of anesthesia (min)	131.8 ± 38.3	136.4 ± 41.0	
Duration of surgery (min)	107.7 ± 36.7	113.6 ± 42.4	
ASA physical status (I/II) (n)	53/65	51/67	
Fluid infusion (mL)	1399 ± 249	1386 ± 222	0.669
Intraoperative Blood loss (mL)	76.7 ± 29.8	79.0 ± 30.0	0.557
Urinary output (mL)	199.2 ± 86.9	205.9 ± 81.2	0.536
Ropivacaine supplements [n (%)]	10 (8.5)	9 (7.6)	0.811

Data are expressed as means ± SD or counts. No significant differences were observed between the two groups. BMI = body mass index, ASA = American Society of Anesthesiologists.

### Primary efficacy outcomes

PCEA requirements were significantly reduced in the parecoxib group at 48 h compared with the placebo group (Table 2). Patients who received parecoxib in the ITT population used a mean number of 2.0 PCEA boluses, and patients in the placebo group used a mean number of 11.5 PCEA boluses, which represents an 83% reduction in PCEA bolus requirements and a 16% reduction in epidural morphine consumption among patients receiving parecoxib compared with those receiving the placebo (*P* < 0.001, Table 2).

**Table 2:** PCEA Bolus Doses and Rescue Analgesics Administered over 48 h.

Parameter	Parecoxib Group	Control Group	MD (95% CI)	*P* Value
ITT population				
No. of patients included in analysis	118	118		
PCEA demanded	0 (0, 3)	7 (2, 15)		<0.001
PCEA delivered	0 (0, 2)	5 (2, 11)		<0.001
Total morphine consumption (mg)	5.01 ± 0.44	5.95 ± 1.29	-0.94 (-1.19, -0.70)*	<0.001
Total ropivacaine consumption (mg)	125.1 ± 11.1	148.7 ± 32.1	-23.6 (-29.7, -17.4)*	<0.001
Amount of tramadol				
0 times	96 (81.4)	82 (69.5)		0.034^&^
1 time	20 (16.9)	32 (27.1)		
2 times	2 (1.7)	4 (3.4)		
Average tramadol consumption (mg)	11.33 ± 24.80	18.60 ± 29.85	-7.27 (-14.31, -0.23)*	0.043
EE population				
No. of patients included in analysis	112	113		
PCEA demanded	0 (0, 3)	8 (3, 15)		<0.001
PCEA delivered	0 (0, 2)	6 (2, 11)		<0.001
Total morphine consumption (mg)	4.97 ± 0.33	5.68 ± 0.94	-0.71 (-0.90, -0.53)*	<0.001
Total ropivacaine consumption (mg)	125.3 ±11.4	149.7 ±32.5	-24.4 (-30.8, -18.0)*	<0.001
Amount of tramadol				
0 times	90 (80.4)	78 (69.0)		0.049^&^
1 time	20 (17.9)	31 (27.4)		
2 times	2 (1.8)	4 (3.5)		
Average tramadol consumption (mg)	11.94 ± 25.32	18.98 ± 30.15	-7.04 (-14.36, 0.28)*	0.059

Data are expressed as means ± SD, medians (lower quartile, upper quartile), percentages (%), or MDs (95% CI).

* Analysis was performed with adjustment of participating centers using the general model.

^&^ Comparison was performed using the Wilcoxon rank-sum test.

MD = mean difference; CI = confidence interval; PCEA = patient-controlled epidural analgesia; ITT = intent-to-treat; EE = efficacy evaluable.

### Secondary efficacy outcomes

**Fig 2:**
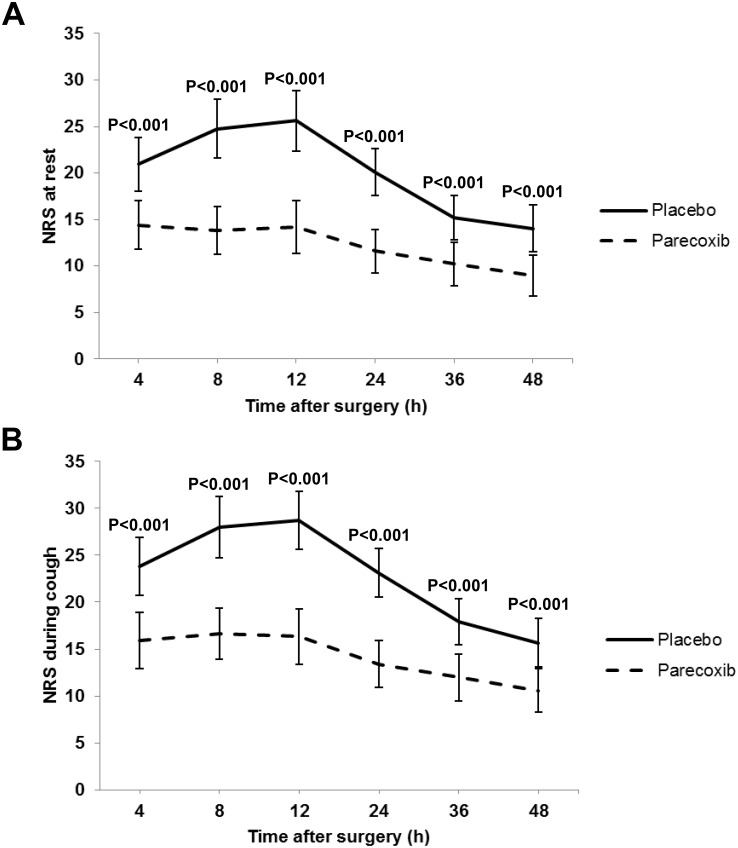
Postoperative numerical rating scores for pain assessments at rest (A) and during coughing (B) at different times after surgery. Patients scored pain using a NRS (0–100 mm, with 0 representing no pain and 100 mm representing the worst imaginable pain). Values are presented as means and 95% confidence interval. Lines extending above and below each plot represent the 95% confidence interval.

The use of parecoxib was associated with significant reductions in pain scores compared with the placebo. NRSs were significantly greater at 4, 8, 12, 24, 36, and 48 h post-surgery in patients receiving placebo compared with parecoxib (*P* < 0.001) (Fig 2). Patients in the parecoxib group required less rescue analgesic tramadol (MD = -11.9%; 95% CI: -22.8–-0.90%; *P* = 0.021) compared with the placebo group (Table 2). Patient satisfaction with their postoperative pain management was significantly higher at 48 h in the parecoxib group compared with the placebo group (100 (8) vs. 90 (8), *P* < 0.001).

### Side effects

Postoperative vomiting was significantly reduced in the parecoxib group. However, the groups were similar with respect to postoperative nausea, pruritus, and the time to the return of bowel function (Table 3). The length of hospitalization was significantly shorter in the parecoxib group compared with the control group (9.50 ± 2.1, 95% CI 9.12~9.88 vs. 10.41 ± 2.6, 95% CI 9.95~10.87, P = 0.003).

**Table 3:** Postoperative Nausea and Vomiting, Pruritus, and Recovery Profiles.

Item	Parecoxib Group (n = 118)	Control Group (n = 118)	*P* Value
Postoperative nausea (n, %)	33 (28.0)	46 (39.0)	0.073
Postoperative vomiting (n, %)	20 (16.9)*	34 (28.8)	0.030
Pruritus (n, %)	28 (23.7)	27 (22.9)	0.878
Time to first flatus (h)	24 (12, 36)	36 (12, 36)	0.302
Time to first bowel movement (h)	48 (36, 60)	48 (24, 72)	0.641

Values are expressed as means ± SD, percentages (%), or medians (lower quartile, upper quartile)

*P < 0.05 compared with the placebo group. (actual P-values specified for all statistically significant variables).

Sedation scores (RSS) and modified Bromage scores were similar between the groups at all time points. Postoperative liver, renal, and coagulation functions did not differ significantly between the two treatment groups (Table 4). No patient in either group experienced wound or cardiovascular complications during hospitalization.

**Table 4:** Postoperative Abnormal Clinical Laboratory Values.

Abnormal Values	Parecoxib Group (n = 112)	Control Group (n = 113)	*P* Value
ALT	10 (8.93%)	8 (7.08%)	0.63
AST	6 (5.36%)	8 (7.08%)	0.78
Cr	1 (0.89%)	2 (1.77%)	1.0
BUN	4 (3.57%)	5 (4.42%)	1.0
PT	5 (4.46%)	3 (2.65%)	0.50
APTT	0	0	1.0
INR	0 (0.00%)	1 (0.88%)	1.0

Data are expressed as percentages (%). No significant differences were observed between the two groups. ALT = alanine aminotransferase, AST = aspartate amino transferase, Cr = creatinine, BUN = blood urea nitrogen, PT = prothrombin time, APTT = activated partial thromboplastin time, INR = international standard ratio.

### EE analysis

Sensitivity analysis of the EE population yielded consistent results for the primary and selected secondary outcomes (Table 2).

## Discussion

This study demonstrated that the perioperative administration of parecoxib reduced the PCEA requirement, epidural morphine consumption, and postoperative vomiting and modestly reduced the length of hospital stay. Parecoxib also improved analgesic effects at rest and during cough and global satisfaction without side effects after abdominal hysterectomy.

CSEA is the most widely used technique for the management of gynecological anesthesia in China [21–23]. Previous studies demonstrated that neuraxial anesthesia exhibited beneficial effects via attenuation of the surgical stress response [24, 25] and postoperative analgesia [26] compared with parenteral opioids.

The epidural catheter was inserted between the L2 and L4 interspaces in this study, which were not located in the middle of the incision segments. Morphine is several-fold less lipophilic than other commonly used opioids, and it is less readily absorbed into the systemic circulation. Morphine is also more widely distributed within the intrathecal space, which provides a better selective spinal effect. A meta-analysis evaluated the efficacy of postoperative epidural analgesia, and epidural morphine was most commonly used (40%), followed by fentanyl (21%) and sufentanil (11%) [26]. Morphine is also the most commonly used epidural analgesic in our centers. Therefore, morphine was chosen as the principal component of the PCEA solution in our study. The recommended dose of epidural morphine is 0.1–1.0 mg/h, followed by a continuous infusion of 3–10 mL/h [27]. The present study used a small basal infusion dose of 0.1 mg/h to minimize side effects, and a low dose (2 mL/h) of the local anesthetic solution was used for additive analgesia.

The combination of NSAIDs and opioids effectively treats postoperative pain via the interruption of nociceptive impulses at central and peripheral sites of the pain transmission pathway, which reduces the need for opioids during the postoperative period [11]. Many studies demonstrated that parecoxib improves postoperative analgesia and decreases PCA opioid consumption by 30–63% following various types of surgery [16, 17, 28–30]. However, few studies evaluated coxibs as adjuvants to PCEA in postoperative pain management [20, 31]. Buvanendran et al. [20] reported that oral rofecoxib decreased PCEA (combination of bupivacaine and fentanyl) bolus doses by 32% in a randomized controlled trial assessing the effect of the perioperative administration of rofecoxib on opioid consumption and outcomes after total knee arthroplasty. Therefore, we speculated that parecoxib would also decrease PCEA bolus doses by 30%. The results of our study are consistent with those of a study by Buvanendran et al. [20]. The present study also revealed that parecoxib 40 mg/12 h was a useful adjuvant to epidural analgesia in patients undergoing gynecological surgery, which allowed a reduction of 83% in PCEA requirements (a reduction of 16% in epidural morphine consumption). The discrepancy between the effect observed by Buvanendran et al. [20], who demonstrated a 32% sparing effect in PCEA requirements, and the findings from this study may result from the various drugs and doses used and the different pathophysiological conditions. We consider the reduction in total morphine consumption (0.94 mg) by parecoxib as clinically relevant because pain scores were reduced, and it resulted in reduced postoperative vomiting and improved patient satisfaction.

Opioid-related side effects of perioperative coxibs are not certain [32]. Patients in the parecoxib group in the present study had an average discharge that was 0.9 days shorter than that of the control group. Better pain control, less morphine consumption, and less PONV may have led to the shorter stays in the parecoxib group. However, few studies reported that reduced hospitalization length was related to NSAIDs [33]. In our study, the length of hospital stay is much longer in both groups compared with other reports [34]. The difference between the greater effect observed in the present study is associated not only with the medical condition but also with the different medical system in China, where patients are hospitalized for preoperative evaluation about 3 days before the operation. Future trials are needed to clarify the effect of coxibs on reductions in hospitalization lengths using a cost-effect analysis.

Apfel et al. [35] showed that the incidence of opioid-induced postoperative nausea and vomiting (PONV) in females was above 40%, and PONV influenced global evaluation ratings. We documented a 41% reduction in vomiting in the parecoxib group with no significant difference in nausea, in contrast to the findings of White et al. [36]. Inoue et al. [37] reported that the incidences of nausea and vomiting were 60% and 35%, respectively, in a study comparing the analgesic efficacy of a morphine and ropivacaine combination for PCEA following gynecological surgery. The routine administration of tropisetron and dexamethasone for antiemetic prophylaxis, avoidance of opioids during the maintenance of anesthesia, and the lower use of epidural morphine and intravenous tramadol clearly contributed to the low incidence of postoperative emetic symptoms in the present study.

Previous studies demonstrated that many conventional preoperative non-selective COX inhibitors increase the risk of perioperative bleeding [38, 39]. Conventional non-selective COX inhibitors interfere with platelet function, but selective COX-2 inhibitors do not affect platelet aggregation or increase intraoperative blood loss [24, 40, 41]. Our data are consistent with these findings because the perioperative administration of 40 mg parecoxib had no significant effects on coagulation function or blood loss, which is consistent with the findings that parecoxib exerts a minimal effect on serum thromboxane and platelet function [13, 41, 42]. However, a significant decrease in hemoglobin concentration during the first 24 hours following skin closure associated with parecoxib (4.3 g⋅dL^− 1^(3.6/4.9) vs. 3.2 g⋅dL^−1^(2.4/5.0)) has been recently reported, while lack of statistical significance in intraoperative blood loss and the total blood loss at 48 hours postoperatively between both groups [43]. An increased incidence of postoperative anemia but lack of statistical significance associated with parecoxib (14.1% vs. 10%) has also been previously reported [44]. Therefore, the effects of selective COX-2 inhibitors on blood loss should be further demonstrated in future trials.

Non-selective NSAIDs inhibit COX-1 and COX-2, which are isoenzymes that are involved in prostaglandin synthesis. The anti-inflammatory effects of non-selective NSAIDs result from the inhibition of COX-2, whereas the harmful gastrointestinal effects of NSAIDs are mediated primarily through the inhibition of COX-1 [45]. Several prospective clinical trials suggested that selective COX-2 inhibitors may be associated with better gastrointestinal tolerance and comparable CV events [46, 47]. Nussmeier et al. [17] demonstrated more comparable upper gastrointestinal and CV events with parecoxib and valdecoxib compared with placebo. This study also revealed no differences in gastrointestinal function and CV events. However, there may be no differences in the incidence of serious vascular events between coxibs and conventional non-selective NSAIDs [48]. Nevertheless, these side effects may not be problematic with the short-term administration of parecoxib to patients with normal renal function and without CV risk factors, as shown in our study.

The limitations of our study include the exclusion of faster-onset opioids (e.g., hydromorphone) in PCEA administration, which are not available in most hospitals in China. Morphine remains the most widely used opioid for pain management because of their advantages of prolonged duration, high analgesic potency, and extended dermatomal level activity. The current study can also be criticized because of the failure to include an active comparable NSAID (e.g., flurbiprofen) or a different type of non-opioid analgesic. Future studies should compare parecoxib with non-selective NSAIDs or a different type of non-opioid analgesic in combination with morphine PCEA after surgery. Thirdly, the study was designed to assess side effects as a secondary outcome measure, and originally powered based on the expected PCEA-sparing effect. Therefore, the sample size may be insufficient to really evaluate risks. Finally, we failed to administer different doses of morphine or parecoxib and examine a dose-response effect. Further trials are required to demonstrate a dose-effect relationship between parecoxib and morphine PCEA.

## Conclusion

The IV administration of parecoxib (40 mg q12 h for 48 h) is an effective adjuvant to epidural analgesia that combines local anesthetic and morphine after gynecological surgery. The short-term use of parecoxib decreased the requirement for epidural morphine, reduced pain scores, resulted in better patient satisfaction, and modestly reduced the length of stay.

## Appendix

### Investigators

Wei-Feng Liu, PhD., M.D., Hai-Hua Shu, PhD. M.D., Ke-Xuan Liu, PhD., M.D., Wen-Qi Huang, M.D., Department of Anesthesiology, The First Affiliated Hospital, Sun Yat-sen University, Guangzhou, China (60 patients); Guo-Dong Zhao, M.D., Department of Anesthesiology, GuangDong General Hospital, Guangzhou, China (60 patients); Jin-Fang Xiao, M.D., Department of Anesthesiology, NanFang Hospital, Guangzhou, China (60 patients); Shu-Ling Peng, PhD., M.D., Department of Anesthesiology, The Second Affiliated Hospital, Sun Yat-sen University, Guangzhou, China (60 patients).

## Supporting Information

S1 Checklist CONSORT 2010. (DOC)

S1 Protocol English copy of trial protocol. (DOC)

S2 Protocol Trial Protocol in Chinese submitted to the hospital ethical. committee. (DOC)
