# Supplementary material for: Effect of Parecoxib as an Adjunct to Patient-Controlled Epidural Analgesia after Abdominal Hysterectomy: A Multicenter, Randomized, Placebo-Controlled Trial
Source: PLoS One. 2016 Sep 13;11(9):e0162589. doi: 10.1371/journal.pone.0162589 (PMC5021366; doi:10.1371/journal.pone.0162589)
Supplement: S1 Protocol — (DOC) [file pone.0162589.s002.doc]

**Study Protocol**

**Efficacy of Parecoxib during Patient-controlled Epidural Analgesia after Abdominal Hysterectomy: A Multicenter, Randomized, Placebo-controlled Trial**

***1. Objective***

This multicenter, randomized, placebo-controlled study evaluated the efficacy and side effects of parecoxib during patient-controlled epidural analgesia (PCEA) after abdominal hysterectomy.

***2. Study design***

***A. multicenter, randomized, double-blinded, placebo-controlled trial***

Study centers ( four centers)

The First Affiliated Hospital, Sun Yat-sen University, Guangzhou, China (60 patients);

GuangDong General Hospital, Guangzhou, China (60 patients);

NanFang Hospital, Guangzhou, China (60 patients);

The Second Affiliated Hospital, Sun Yat-sen University, Guangzhou, China (60 patients).

***B. The inclusion criteria***

Women with an American Society of Anesthesiologists physical status class I-II, aged 18-64 years, who were undergoing abdominal hysterectomy (with or without oophorectomy) under combined spinal-epidural anesthesia (CSEA) were assessed for study eligibility. Written informed consent was obtained prior to the day of surgery.

***C. The exclusion criteria***

Contraindications for CSE placement; known allergy, sensitivity, or contraindication to opioid and non-opioid analgesic drugs; history of bleeding disorders; peptic ulceration; or anticoagulant use within the past month; drug or alcohol abuse; current pregnancy or breastfeeding; and lack of ability to understand the use of pain assessment scales and the PCA device. Patients with asthma or bronchospasm who required treatment with glucocorticoids, poorly controlled hypertension or diabetes, a chronic or acute renal or hepatic disorder, or inflammatory bowel disease were also excluded. Patients were also excluded if they had used long-acting NSAIDs in the 4 days prior to the first dose of study medication or if they had taken antidepressants, narcotic analgesics, antihistamines, anxiolytics, hypnotics, sedatives, NSAIDs, or corticosteroids up to 24 h before administration of the study medication.

***D. Grouping***

Patients were randomized 1:1 to receive either 40 mg of parecoxib sodium (Pfizer Ltd, Pharmacia and Upjohn Company) (Group Parecoxib) IV or saline (Dazhong Pharmaceuticals Limited, China) (Group Control) at the same volume prior to the initial incision. Thereafter, patients in the parecoxib group received 40 mg of parecoxib IV every 12 h, and patients in the control group received 2 mL of saline every 12 h for 48 h. Post-anesthesia care unit nurses who were blinded to the study prepared the intervention drugs.

***E. Study procedures***

The patient was placed in the lateral position to receive the CSEA. The back was sterilely prepared and draped, and 1% lidocaine was injected subcutaneously for local anesthesia into the lower lumbar vertebral interspace between L2 and L4. The spinal component, which consisted of 10-15 mg of 0.5% or 0.75% hyperbaric bupivacaine, was infused into the intrathecal space. If the spinal block proved insufficient for surgery, epidural 1% ropivacaine supplements (maximum of 8 mL) were administered as clinically indicated to maintain a T6 level of sensory blockade. Tropisetron (0.1 mg/kg) and dexamethasone (0.1 mg/kg) were routinely used for antiemetic prophylaxis 30 min before the end of surgery.

A mixture of 6 mL of 0.25% ropivacaine and 2 mg morphine was administered epidurally 30 min prior to the end of surgery. PCEA was initiated using 0.125% (1.25 mg/mL) ropivacaine and 0.005% (0.05 mg/mL) morphine with a basal infusion rate of 2 mL/h, a demand dose of 2 mL, and a 15-min lockout. Patients were instructed prior to surgery to use the PCEA mode at their discretion to maintain a numerical rating score (NRS) less than 3. Patients received 1 mg/kg of intravenous tramadol if the NRS was 4 or greater.

***F. Primary and secondary outcome measures***

The primary end point of this study was the quantification of the PCEA-sparing effect of a multimodal approach that contained parecoxib compared with a standard PCEA approach that did not contain parecoxib in patients undergoing abdominal hysterectomy. The number of PCEA bolus doses and total morphine consumption administered by the patient for 48 h postoperatively were recorded.

Secondary outcomes included pain intensity, the need for rescue analgesics, global satisfactory patient evaluation, and side effects, such as nausea, vomiting, pruritus, sedation, motor block in the lower extremities, and the time to the passage of flatus and the first bowel movement. Pain intensity was recorded on a 100-mm NRS ranging from 0 (no pain) to 100 (worst pain imaginable) and measured at rest and during activity. Pain during activity was elicited by asking the patient to take a deep breath followed by a forceful cough. A pain assessment was obtained immediately after the cough. A global satisfactory evaluation (using a 100-point scale from 1 = highly dissatisfied to 100 = highly satisfied) was obtained from the patient 48 h after skin closure. Assessment of sedation was performed using the Ramsay Sedation Scale (RSS). Motor block was assessed using a modified Bromage scale. Blood loss, length of hospitalization, and postoperative cardiovascular (CV) events were also recorded. Discharge was assessed by the surgeons according to the following discharge criteria: a) pain controlled with oral analgesics; b) stable vital signs; c) afebrile; d) passing flatus; e) incision clean, dry, and intact; and f) full diet tolerated. Pain scores, vital signs (measured using standard monitors during the first 24 h and a portable blood pressure monitor and pulse oximeter 36 h and 48 h after skin closure), the RSS, recovery of gastrointestinal function, and side effects were assessed at 4, 8, 12, 24, 36, and 48 h after skin closure by an investigator who was blinded to the treatment groups.

Blood samples were drawn before and 48 h after surgery to determine liver (alanine aminotransferase and aspartate amino transferase) and renal (plasma creatinine and blood urea nitrogen concentration) function. Prothrombin time, active partial thromboplastin time, and international standard ratio (INR) were also assessed.
