# Supplementary material for: Effect of Parecoxib as an Adjunct to Patient-Controlled Epidural Analgesia after Abdominal Hysterectomy: A Multicenter, Randomized, Placebo-Controlled Trial
Source: PLoS One. 2016 Sep 13;11(9):e0162589. doi: 10.1371/journal.pone.0162589 (PMC5021366; doi:10.1371/journal.pone.0162589)
Supplement: S2 Protocol — (DOC) [file pone.0162589.s003.doc]

**帕瑞昔布钠复合硬膜外吗啡术后镇痛**

**应用于妇科手术的效果和安全性**

1．研究内容

观察帕瑞昔布钠复合硬膜外吗啡术后镇痛应用于妇科手术的效果和安全性。

2．研究设计

A. 前瞻性、多中心（4中心）、随机、双盲、安慰剂对照、平行分组研究。

研究中心：广东省人民医院

南方医科大学附属南方医院

中山大学附属第二医院

中山大学附属第一医院（PI单位）

B. 纳入标准：

a. 18～64岁、择期腰硬联合麻醉下妇科开腹手术

b. ASA分级Ⅰ-Ⅱ级

c. 同意参加本研究并签署知情同意书的患者

C. 排除标准：

a. 既往有心绞痛或充血性心力衰竭、心肌梗死、冠状动脉血管重建术、冠状动脉搭桥、中风或一过性缺血发作、不能控制的高血压或糖尿病、需要激素治疗的哮喘、肝脏疾病、肾脏疾病、炎性肠病、出血性疾病等疾病的患者。

b. 对NSAIDs、阿片类、局麻药或磺胺类药物过敏，或存在明显的临床或实验室异常病史因而禁忌使用帕瑞昔布或阿片类药物的患者。c. 近期有酗酒和镇痛药物滥用史，或强效阿片类药物和抗抑郁药物治疗史的患者。

d. 如果患者已服用NSAIDs，则需至少4天的洗脱期，并提供弱阿片类药物曲马多作为替代药物。

e. 不能合作的患者，或不能理解和执行VAS评分的患者。CSEA禁忌者。

D. 病人分组：符合条件的病人240例（每个中心60例）随机分为帕瑞昔布钠40mg组（P组）及安慰剂组（C组）。

E. 研究药物: 由非研究人员进行随机分组并配备研究药物和镇痛泵。用生理盐水2 ml将帕瑞昔布钠40 mg溶解成与生理盐水外观一致的无色透明溶液，或对照药物生理盐水2 ml，供研究人员使用。

F. 麻醉方法：于L2/3或L3/4进行腰硬联合麻醉，予0.5%或0.75%布比卡因10～15 mg 蛛网膜下腔注入。麻醉平面控制在T6~T8，每2h或麻醉阻滞平面不全时于硬膜外腔推注1%罗哌卡因（耐乐品）6~8ml。术中镇静可予咪达唑仑(midazolam) 0.05mg/kg或丙泊酚<2~6mg/kg/h。

术中应适当补充血容量，一般术中均采用复方乳酸溶液(RL) 即可，腰麻药物注射后应补充RL 500~1000 mL, 术中根据循环状况给予足够RL。围术期不采用任何人工胶体溶液，输血严格遵循输血指征；红细胞压积（Hct） <21% 、血红蛋白（Hb）<70 g/L时开始输血，并达到Hb>100 g/L即可，术中建议及时检测观察Hb。围术期严格遵循输血浆指征，止血药等仅在必要时应用。

确保手术患者围术期生命安全和麻醉效果，中小型手术, 不可过度治疗,不需要进行动、静脉穿刺(病情需要除外)。

所有麻醉后术毕的患者必须送入麻醉恢复室（PACU），并观察至达到送出PACU的标准。妇科手术术后呕吐率高，常规使用二联止吐药（地塞米松5mg +5-HT3 受体阻滞剂）。

G. 给药方案：PCEA

镇痛泵药液配方：50ug/ml 吗啡+ 0.125%罗哌卡因（耐乐品）。使用电子镇痛泵，设置持续剂量为2 mL/h , 患者自控加药剂量每次为2 mL , 自控间隔时间为15min。

P组：手术开始前，静脉注射首剂帕瑞昔布钠（特耐 parecoxib）40 mg；手术结束前30 min硬膜外腔给予镇痛首量2mg吗啡+0.125%罗哌卡因（耐乐品）6ml。

C组：手术开始前，静脉注射首剂生理盐水2ml；手术结束前30 min硬膜外腔给予镇痛首量2mg吗啡+0.125%罗哌卡因（耐乐品）6ml；术毕进行VAS评分。

首剂药物后12h、24h和36h，静脉注射试验药物(帕瑞昔布钠或生理盐水)。

H. 观察项目：1.帕瑞昔布钠对术后吗啡节俭作用的有效性 (1) 首剂药物后2、4、6、9、12、18、24、36和48 h的疼痛强度(100-mm VAS评分)；(2) 作为镇痛的补救措施（VAS>4时给予曲马多1mg/kg）：采用其他镇痛药物的次数和剂量，如果在给予研究药物1 h内给予其他镇痛的补救措施，此病人将不进入疗效分析；(3) 首剂药物后48 h病人对镇痛的满意度；(4) 改良Bromage运动阻滞评分：0分，无运动阻滞（可充分屈髋、膝、踝关节）；1分，不能直腿抬起（仅能屈膝、踝关节）；2分，不能屈膝（仅能屈踝关节）；3分，膝、踝关节均不能屈； 2．帕瑞昔布钠的安全性 (1)给药前和完成研究后的肌酐、BUN、ALT、AST、总胆红素、血红蛋白、血小板、凝血酶原时间(PT)和激活部分促凝血酶原激酶时间(APTT)，检查结果超过正常值为异常；(2)给药前和完成研究时卧位的生命体征：血压、心率、体温和呼吸频率；(3)给药后出现的任何有关的不良反应及PCEA并发症。

3. 年度研究计划

起止时间及主要工作内容

2009年6月-2010年4月，完成资料收集

2010年5月-2010年6月，完成所有研究，进行数据统计，撰写论文。
